# Preliminary Assessment of Hurricane Harvey Exposures and Mental Health Impact

**DOI:** 10.3390/ijerph15050974

**Published:** 2018-05-13

**Authors:** Rebecca M. Schwartz, Stephanie Tuminello, Samantha M. Kerath, Janelle Rios, Wil Lieberman-Cribbin, Emanuela Taioli

**Affiliations:** 1 Department of Occupational Medicine, Epidemiology and Prevention, Hofstra Northwell Health School of Medicine, Great Neck, NY 11021, USA; Rschwartz3@northwell.edu (R.M.S.); Skerath@northwell.edu (S.M.K.); 2 Department of Population Health Science and Policy and Institute for Translational Epidemiology, Icahn School of Medicine at Mount Sinai, New York, NY 10029, USA; Stephanie.Tuminello@mssm.edu (S.T.); wil.lieberman-cribbin@icahn.mssm.edu (W.L.C.); 3 Center for Disaster Health, Trauma and Resilience; Mount Sinai, Stony Brook University, Northwell Health, Stony Brook, NY 11794, USA; 4 Center for Biomedical Science, Feinstein Institute for Medical Research, Manhasset, NY 11030, USA; 5 The University of Texas School of Public Health, Houston, TX 75235, USA; Janelle.Rios@uth.tmc.edu

**Keywords:** extreme weather event, disaster, post-traumatic stress disorder, emergency response, epidemiology

## Abstract

Hurricane Harvey made landfall in Houston, Texas on 25 August 2017, the psychological and physical effects of which are still unknown. We assessed hurricane exposure and the immediate mental health needs of the population to define public health priorities for a larger epidemiological study. Convenience sampling was used to recruit participants (*n* = 41) from the greater Houston area aged ≥18 years. Participants completed a questionnaire about demographics, hurricane exposures, and physical/mental health. Post-Traumatic Stress Disorder (PTSD) was measured with the Post-Traumatic Stress Disorder Checklist-S (PCL-S; a score ≥30 indicated probable PTSD symptoms). The Patient Health Questionnaire-4 (PHQ-4) was used to assess symptoms of depression and generalized anxiety disorder. The average PTSD score was 32.9 (SD = 17.1); a total of 46% of participants met the threshold for probable PTSD. Increased overall hurricane exposure (adjusted odds ratio (OR_adj_) 1.42; 95% confidence interval (CI): 1.06–2.05) and property-related exposure (OR_adj_ 1.53; 95% CI: 1.07–2.18) were both statistically significantly associated with increased odds of probable PTSD symptoms. A perception of chemical/toxin exposure due to Hurricane Harvey was reported by 44% of participants. A higher number of personal or property exposures were associated with greater mental health symptoms three weeks post-hurricane. This work has implications for the ongoing response to Hurricane Harvey and for assessing the immediate needs of the population.

## 1. Introduction

At 10 PM on 25 August 2017, Hurricane Harvey hit the coast of Texas as a Category 4 hurricane with wind speeds greater than 58 m/s. Between landfalls over a four day period, Hurricane Harvey brought torrential rains totaling about 50 inches at Bush International Airport in Houston, Texas [1]. This storm was particularly devastating to the greater Houston area, a city with 2.3 million people. Various sources and media outlets indicated 30,000 residents displaced, $70–$170 billion in property damage, and half-million vehicles and untold structures flooded.

Hurricane exposure has been shown to have a profound impact on the mental health of affected residents, leading to increased symptomology of stress, anxiety, depression and Post-Traumatic Stress Disorder (PTSD) [2,3,4]. Being displaced because of the storm, especially to a temporary shelter, has been shown to exacerbate these mental health issues, possibly because of the perceived decrease in social support associated with displacement [5,6,7,8,9].

During Hurricane Harvey there was also the potential for chemical exposures due to numerous chemical plants, natural gas and oil refineries present in the Houston area. More than 50 Environmental Protection Agency (EPA) Superfund sites (sites of previous contamination) are located in the area, some of which were flooded and potentially contaminated the flood waters in their surroundings [10]. Further, anecdotal exposure to mold and sewage bacteria in flooded streets was widely reported. Exposures related specifically to chemicals can have both short and long-term negative impacts on mental health [11,12,13,14]. Previous research indicates an indirect impact of chemical disasters on mental health. After the Deepwater Horizon Oil Spill, participants who experienced job loss and disruption in social and occupational functioning as a result of the oil spill were more likely to experience anxiety and depression [11,12]. A study of South Carolina residents following a chlorine gas disaster indicated that physical health symptoms were significantly associated with post-traumatic stress, suggestive of an interaction between physical and mental health during exposures to chemicals and toxins [13]. Similarly, dampness and mold were associated with depression, but the association was mediated by perception of control over one’s home and by physical health [14]. Another issue related to chemical exposures is cognitive bias; the perceived odor and cognitive expectations of a chemical can negatively affect how an individual responds to that chemical [15]. The impact of chemical exposures on the physical and mental health of an affected population becomes even more pronounced when combined with other psychological stressors related to hurricanes, such as escaping flooded homes, finding shelter and temporary housing, or replacing damaged property [16]. Anecdotal reports from healthcare providers hint at these psychological and physical effects; however, the full effects of both the actual chemical contamination and the indirect effects of perceived exposures due to Hurricane Harvey are still being elucidated.

We conducted a preliminary assessment of the initial psychological impact of Hurricane Harvey in an effort to assess the immediate mental health needs of the population and to define public health priorities for a larger epidemiological study. Proximity in time to Hurricane Harvey was an important consideration in our research planning as the immediate weeks following a natural disaster are typically when the mental health impacts are arguably the most severe [17,18]. To our knowledge we were one of the first research teams to reach the Houston area following Hurricane Harvey.

## 2. Materials and Methods

Our research team was in Houston less than 3-weeks after Hurricane Harvey made landfall, which was the quickest our team could mobilize, and immediately began surveying affected residents. Convenience sampling was used to recruit participants from heavily affected areas including the George R. Brown convention center which, in the immediate aftermath of Hurricane Harvey, was converted into a temporary shelter. Participants who were ≥18 years old and had resided within the greater Houston area during the hurricane were eligible and were given a $10 Target gift card as reimbursement for their time. Most found out about the study through word of mouth. Approval for this study was given by the internal review board of the Icahn School of Medicine at Mount Sinai on 9/11/2017 (HS#: 15-00513) as a modification of our existing IRB created for researching the effects of Hurricane Sandy.

### 2.1. Hurricane Exposures

Study participants were consented and completed a questionnaire about demographics, hurricane exposures, and physical/mental health before and after Hurricane Harvey, modelled on studies we conducted on Sandy affected populations [2,19]. Personal hurricane exposures were those that directly affected the participant or their family, and property related exposures were exposures related to the level of personal property affected and the resulting financial hardship (Supplementary Table S1) [19]. The grouping of an exposure item into either the Personal or Property-related category was based on the results of a Principal Components Analysis conducted by Schwartz et al. [19]. There was a total of 16 personal exposures and 14 property exposures measured. A “total exposure score” was also generated by summing affirmative answers (1 = Yes) to each of the 30 items (personal and property) on the hurricane exposure scale. Displacement, one of the personal exposure items, was also examined separately to determine the mental health correlates of displacement specifically.

Participants also reported exposure to specific chemicals and toxins (Yes/No) known to be associated with Harvey, such as debris, mold, petroleum, and chemical emissions (e.g., carbon monoxide).

### 2.2. Mental Health Outcomes

The primary outcomes were mental health symptoms of anxiety, depression and PTSD. PTSD symptoms were examined using the Post-Traumatic Stress Disorder Checklist-S (PCL-S), a 17-item self-report measure that asked about PTSD symptoms specific to Hurricane Harvey. A score ≥30 was considered indicative of probable PTSD symptoms. The Patient Health Questionnaire-4 (PHQ-4) was used to assess symptoms of depression and of generalized anxiety disorder; a score ≥3 was considered indicative of probable depression or anxiety.

### 2.3. Statistical Analysis

Fisher’s Exact Test or Wilcoxon Rank Sum and multivariate logistic regression were used to evaluate associations between hurricane exposures and mental health. Having probable PTSD, anxiety or depression was the primary outcome in the logistic regression statistical models, and was treated as a dichotomous variable (yes or no) based on the clinically relevant cutoffs described above. The number of personal, property-related or chemical exposures was used as a continuous variable to investigate how an increase in any type of hurricane exposure affected mental health. Logistic models were adjusted for covariates including age, gender and a prior history of a mental health condition, including anxiety disorder, depression, PTSD, schizophrenia, bipolar disorder, substance abuse disorders (alcohol or prescription drug related) or some other mental health disorder that was diagnosed by a physician. We chose to include these covariates because of their perceived biological significance, which is supported by the literature [20]. Health insurance status was also included in the adjusted models as it was found to be statistically associated with both PTSD and depression in the bivariate analysis (data not shown), and because it acts as a proxy for socioeconomic status. Though other variables such as race, ethnicity and education might still have a confounding effect, our data represents only a small preliminary assessment of the affected Houston population and we did not have sufficient sample size to run logistic models with adjustment for all these covariates. Data analysis was performed using SAS (SAS Institute, Cary, NC, USA, V9.4) software.

## 3. Results

The study sample consisted of 41 participants who had lived in the greater Houston area during Hurricane Harvey. The majority was female (56%) US born (78%), and had some type of health insurance (78%). The study cohort was mostly White (34%), Black (32%) and Hispanic (20%); the remaining participants self-identified as American Indian, Pacific Islander, Asian or Other. The majority of participants had attended at least some college (76%). The mean age was 44 years (SD = 10 years), with a mean household size of 2.4 people (Table 1).

### 3.1. Hurricane Exposures

**Figure 1:**
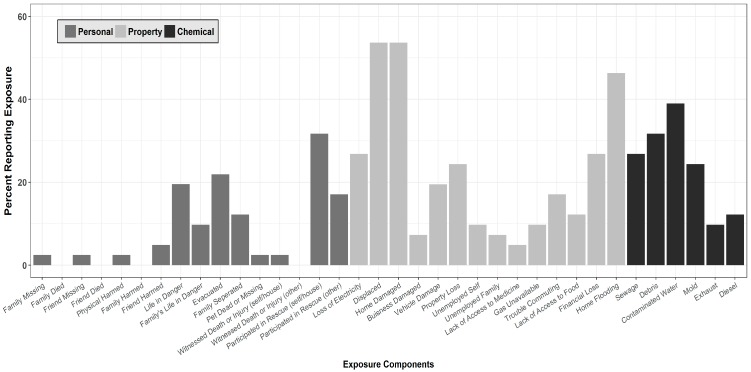
Hurricane Exposure Assessment (*n* = 41). The mean number of overall hurricane exposures was 4.19 (SD = 4.56; range 0–30).

Most participants (88%) reported experiencing some type of exposure to Hurricane Harvey. Overall, 34% of study participants reported at least one type of Personal exposure, while 61% reported at least one type of Property-related exposure. The most commonly reported personal exposure items were assisting in rescue efforts (31.7%) and being evacuated from their homes (22.0%). For Property-related exposure, participants most commonly reported being displaced (53.7%), having their homes damaged (53.7%), and having flooding in their homes (46.3%; Figure 1).

A perception of chemical/toxin exposure due to Hurricane Harvey was also reported by a large number of participants (44%): 39% reported being exposed to dirty or contaminated flood water, 27% to sewage, and 12% to oil leaks. Participants in this study also reported being exposed to mold (24.4%) and debris (31.7%; Figure 1).

### 3.2. Mental Health Symptomology

PCL scores ranged from 17–71, with a mean score of 32.9 (SD = 17.1); 46% of participants met the threshold for probable PTSD symptoms. As determined by the PHQ-4, 53.7% of participants experienced anxiety symptoms and 39.0% experienced depression symptoms post-Harvey.

After adjustment, increased overall hurricane exposure (adjusted odds ratio (OR_adj_) 1.42; 95% confidence interval (CI): 1.06–2.05) and property-related exposure (OR_adj_ 1.53; 95% CI: 1.07–2.18) were both statistically significantly associated with an increased odds of probable PTSD symptoms. Further, being displaced during the hurricane was also significantly associated with increased odds of probable PTSD symptoms (OR_adj_ 12.50; 95% CI: 1.43–108.92).

After adjustment, an increase in chemical/toxin exposure (OR_adj_ 1.96; 95% CI: 1.16–3.32), overall hurricane exposure (OR_adj_ 1.50; 95% CI: 1.09–2.06), and property-related hurricane exposure (OR_adj_ 1.60; 95% CI: 1.13–2.28) were all statistically significantly associated with increased odds of probable anxiety.

In terms of probable depression symptoms, only the relationship with displacement remained significant after adjustment (OR_adj_ 15.76; 95% CI: 1.22–203.06) (Table 2).

## 4. Discussion

### 4.1. Hurricane Exposure and Mental Health

Preliminary results indicate that increased Hurricane Harvey exposure may have had a significant impact on the mental health of Houston residents. The reported number of personal or property exposures was positively associated with mental health symptoms, particularly PTSD symptoms, three weeks post-hurricane. Further, consistent with our previous work on displacement during Hurricane Sandy, the current study indicated that displacement was associated with an increased risk of PTSD and depression symptoms [19,21].

The data also shows an association between perceived chemical/toxin hurricane exposure and PTSD and anxiety symptoms. The results support previous research reporting that exposures related specifically to chemicals have negative short and long-term impacts on mental health [11,12,22]. However, the interaction between physical and mental health following exposure to toxins/chemicals after a natural disaster is still largely unknown and should be the focus of further studies, including a comparison of objective versus perceived measurement of chemical exposure.

### 4.2. Strengths and Limitations

This study was based on a small convenience sample; it is cross-sectional, and data interpretation was correlational with no causal inferences made. However, to our knowledge, this represents the first reported assessment of Hurricane Harvey’s impact on Houston residents’ mental health. It incorporated validated instruments to investigate the degree of hurricane exposure and its impact on mental health symptoms. It should be noted that it is not possible to receive a true diagnosis of PTSD only three weeks after a traumatic event, however the PCL-S is used to assess symptoms and is not used to make a true diagnosis. Further, it is possible that the PTSD scale is assessing acute stress symptoms, as opposed to potential PTSD symptoms, given the close proximity to the hurricane. Recall bias, a potential limitation of this study design, was minimized by the quick mobilization of the research team.

This study’s proximity in time to the hurricane caused limitations to our recruitment. Being in Houston so soon after the hurricane meant that we faced physical barriers such as flooded and otherwise inaccessible roads. However, this also enabled us to recruit participants while they were still displaced to a shelter. The need to seek a temporary shelter most likely compounds the hurricane-related mental health symptoms caused or exacerbated by the storm [2,6,23]. Moreover, having a pre-existing mental health condition has been linked to greater mental health symptomology post-hurricane, and those seeking temporary shelter are often unable to retrieve their necessary psychiatric medication during an evacuation [18,24]. The fact that we do not see a similar statistically significant relationship in our study between pre-existing mental health disorders and mental health symptoms post-hurricane can most likely be attributed to the study’s small sample size. Research directly after the hurricane is necessary to assess the mental health needs of displaced residents.

### 4.3. Future Research Implications

The lessons gained from conducting this preliminary assessment of Houston residents immediately after Hurricane Harvey has been instrumental in our future research endeavors. One of the most significant research barriers we faced during this initial assessment was receiving permission to be at the shelter as a research group. We were unsure who the designated decision-maker at the shelter was and what protocol we needed to follow to allow us access to the shelter for research purposes. Though we were able to overcome these obstacles, it speaks to the great need to have community partnerships and infrastructure in place prior to disasters [25]. After Hurricane Sandy, community engagement with those in the Rockaway area of New York allowed us to link those affected by the hurricane to local mental health resources including an existing mental health and substance abuse treatment center in the area. Hopefully, with guidance from this assessment and future research, similar programs will be able to be organized in Houston.

## 5. Conclusions

This assessment of the hurricane’s initial impact on mental health lays the groundwork for future research. It is clear that residents were greatly impacted by the hurricane and that the types of exposures they suffered were vast and variable. These findings have implications both for the ongoing response to Hurricane Harvey and the emergency preparedness community as a whole. Literature points to the stability of PTSD over time among communities exposed to natural disasters, with rates as high as 30–40% [4], indicating the need to address symptoms as early as possible and to provide long-term support for those affected.

## Figures and Tables

**Table 1:** Characteristics of the population under study.

Demographics	*N* = 41
*Gender*	
Female	23 (56.1%)
Male	18 (41.9%)
*Ethnicity*	
White	14 (35.0%)
Black	13 (32.5%)
American Indian	1 (2.5%)
Hispanic	8 (20.0%)
Pacific Islander/Asian	4 (10.0%)
*US Born*	
No	9 (22.0%)
Yes	32 (78.0%)
*Education*	
<High School Degree	3 (7.9%)
High School Degree	6 (15.8%)
Some College	8 (21.0%)
College Degree	8 (21.1%)
Post-Graduate Degree	13 (34.2%)
*Health Insurance*	
No	9 (22.0%)
Yes	32 (78.0%)
*Mental Health Condition Prior to Harvey*	
No	29 (70.7%)
Yes	12 (29.3%)
*Age* (years)	44.2 ± 10.3
*Household Size* (# of people)	2.4 ± 1.1

Frequency missing: Ethnicity (*n* = 1), Education (*n* = 3), Age (*n* = 3), Household Size (*n* = 1).

**Table 2:** Association between Hurricane Harvey exposure and mental health symptoms.

Exposure	PTSD (PCL ≥ 30) Mean (SD)			Anxiety (PHQ-4 ≥ 3) Mean (SD)			Depression (PHQ-4 ≥ 3) Mean (SD)		
	No	Yes	OR_adj__*_ (95% CI)	No	Yes	OR_adj__*_ (95% CI)	No	Yes	OR_adj__*_ (95% CI)
Chemical/Toxin	0.77 (1.45)	2.21 (2.35) ^^^	1.45 (0.95–2.21)	0.53 (1.12)	2.23 (2.31) ^+^	1.96 (1.16–3.32)	1.04 (1.85)	2.06 (2.21)	1.36 (0.94–1.96)
Hurricane overall	2.36 (2.32)	6.95 (5.29) ^^^	1.42 (1.06–2.05)	2.36 (2.34)	6.33 (5.22) ^^^	1.50 (1.09–2.06)	3.72 (4.66)	5.69 (4.25) ^@^	1.10 (0.93–1.31)
Hurricane Personal	0.68 (0.84)	2.00 (2.26) ^#^	2.29 (0.92–5.71)	0.79 (0.92)	1.73 (2.19)	1.97 (0.92–4.27)	1.20 (1.68)	1.44 (1.93)	1.10 (0.72–1.68)
Hurricane Property	1.68 (1.94)	4.95 (3.44)	1.53 (1.07–2.18)	1.58 (1.92)	4.59 (3.39) ^^^	1.60 (1.13–2.28)	2.52 (3.28)	4.25 (2.74) ^+^	1.18 (0.93–1.51)
Displacement (%)									
No	17 (89)	2 (11) ^^^	1 (ref)	13 (68)	6 (32) ^^^	1 (ref)	16 (84)	3 (16) ^^^	1 (ref)
Yes	5 (23)	17 (77)	12.50 (1.43–108.92)	6 (27)	16 (73)	6.66 (0.922–48.09)	9 (41)	13 (59)	15.76 (1.22–203.06)

* adjusted for age (continuous), gender, health insurance status and previous history of mental health conditions; *n* = 39, 2 people were missing age; ^^^ *p* < 0.01; ^#^ *p* = 0.04; ^+^ *p* = 0.02; ^@^ *p* = 0.03.

## Supplementary Materials

The following are available online at http://www.mdpi.com/1660-4601/15/5/974/s1, Table S1: Personal vs. Property Hurricane Exposure Items.

## References

[1] CNN B.G. CNN Design: Lansing Cai Harvey’s Devastating Impact by the Numbers. http://www.cnn.com/2017/08/27/us/harvey-impact-by-the-numbers-trnd/index.html.

[2] Schwartz R.M., Sison C., Kerath S.M., Murphy L., Breil T., Sikavi D., Taioli E. (2015). The impact of Hurricane Sandy on the mental health of New York area residents. Am. J. Disaster Med..

[3] Cerdá M., Bordelois P.M., Galea S., Norris F., Tracy M., Koenen K.C. (2013). The course of posttraumatic stress symptoms and functional impairment following a disaster: What is the lasting influence of acute versus ongoing traumatic events and stressors?. Soc. Psychiatry Psychiatr. Epidemiol..

[4] Neria Y., Nandi A., Galea S. (2008). Post-traumatic stress disorder following disasters: A systematic review. Psychol. Med..

[5] Schwartz R.M., Liu B., Lieberman-Cribbin W., Taioli E. (2017). Displacement and mental health after natural disasters. Lancet Planet. Health.

[6] Lê F., Tracy M., Norris F.H., Galea S. (2013). Displacement, county social cohesion, and depression after a large-scale traumatic event. Soc Psychiatry Psychiatr. Epidemiol..

[7] Schwartz R.M., Rasul R., Kerath S.M., Watson A.R., Lieberman-Cribbin W., Liu B., Taioli E. (2018). Displacement during Hurricane Sandy: The impact on mental health. J. Emerg. Manag..

[8] Fullilove M.T. (1996). Psychiatric implications of displacement: Contributions from the psychology of place. Am. J. Psychiatry.

[9] Wadsworth M.E., Santiago C.D., Einhorn L. (2009). Coping with displacement from Hurricane Katrina: Predictors of one-year post-traumatic stress and depression symptom trajectories. Anxiety Stress Coping.

[10] CNBC Hurricane Harvey Rains Flood Toxic Superfund Sites in Texas. https://www.cnbc.com/2017/09/03/hurricane-harvey-rains-flood-toxic-superfund-sites-in-texas.html.

[11] Grattan L.M., Roberts S., Mahan W.T., McLaughlin P.K., Otwell W.S., Morris J.G. (2011). The early psychological impacts of the Deepwater Horizon oil spill on Florida and Alabama communities. Environ. Health Perspect..

[12] Osofsky H.J., Osofsky J.D., Hansel T.C. (2011). Deepwater horizon oil spill: Mental health effects on residents in heavily affected areas. Disaster Med. Public Health Prep..

[13] Clark K.A., Chanda D., Balte P., Karmaus W.J., Cai B., Vena J., Lawson A.B., Mohr L.C., Gibson J.J., Svendsen E.R. (2013). Respiratory symptoms and lung function 8–10 months after community exposure to chlorine gas: A public health intervention and cross-sectional analysis. BMC Public Health.

[14] Shenassa E.D., Daskalakis C., Liebhaber A., Braubach M., Brown M. (2007). Dampness and Mold in the Home and Depression: An Examination of Mold-Related Illness and Perceived Control of One’s Home as Possible Depression Pathways. Am. J. Public Health.

[15] Dalton P., Wysocki C.J., Brody M.J., Lawley H.J. (1997). The influence of cognitive bias on the perceived odor, irritation and health symptoms from chemical exposure. Int. Arch. Occup. Environ. Health.

[16] Peek M., Cutchin M., Freeman D., Stowe R., Goodwin J. (2009). Environmental hazards and stress: Evidence from the Texas City Stress and Health Study. J. Epidemiol. Community Health.

[17] Flory K., Kloos B., Hankin B.L., Cheely C.A. (2008). Clinical Research After Catastrophic Disasters: Lessons Learned From Hurricane Katrina. Prof. Psychol. Res. Pract..

[18] Ochi S., Hodgson S., Landeg O., Mayner L., Murray V. (2014). Disaster-driven evacuation and medication loss: A systematic literature review. PLoS Curr..

[19] Schwartz R., Liu B., Sison C., Kerath S.M., Breil T., Murphy L., Taioli E. (2016). Study Design and Results of a Population-Based Study on Perceived Stress Following Hurricane Sandy. Disaster Med. Public Health Prep..

[20] Schwartz R.M., Gillezeau C.N., Liu B., Lieberman-Cribbin W., Taioli E. (2017). Longitudinal Impact of Hurricane Sandy Exposure on Mental Health Symptoms. Int. J. Environ. Res. Public Health.

[21] Rebecca M., Schwartz P., Patricia Rothenberg B.A., Samantha M. Kerath M.S., Bian Liu P., Emanuela Taioli M.D. (2016). The lasting mental health effects of Hurricane Sandy on residents of the Rockaways. J. Emerg. Manag..

[22] Ginsberg J.P., Holbrook J.R., Chanda D., Bao H., Svendsen E.R. (2012). Posttraumatic stress and tendency to panic in the aftermath of the chlorine gas disaster in Graniteville, South Carolina. Soc. Psychiatry Psychiatr. Epidemiol..

[23] Fussell E., Lowe S.R. (2014). The impact of housing displacement on the mental health of low-income parents after Hurricane Katrina. Soc. Sci. Med..

[24] Sullivan G., Vasterling J.J., Han X., Tharp A.T., Davis T., Deitch E.A., Constans J.I. (2013). Preexisting Mental Illness and Risk for Developing a New Disorder After Hurricane Katrina. J. Nerv. Ment. Dis..

[25] Springgate B.F., Wennerstrom A., Meyers D., Allen C.E., Vannoy S.D., Bentham W., Wells K.B. (2011). Building community resilience through mental health infrastructure and training in post-Katrina New Orleans. Ethn. Dis..

