# Supplementary material for: Preliminary Assessment of Hurricane Harvey Exposures and Mental Health Impact
Source: Int J Environ Res Public Health. 2018 May 13;15(5):974. doi: 10.3390/ijerph15050974 (PMC5982013; doi:10.3390/ijerph15050974)
Supplement: Supplementary file 1 [file ijerph-15-00974-s001.pdf]

Supplementary Table 1: Personal vs. Property Hurricane Exposure Items.

**Type of Hurricane Exposure**	**Self-Reported Hurricane Harvey Experience**
Personal Hurricane Exposure	Family member missing<br>Family member died<br>Friend missing<br>Friend died<br>You were physically harmed<br>Someone in your family was physically harmed<br>Friend was physically harmed<br>Your life was in danger<br>The life of family member(s) was in danger<br>Evacuated quickly with no time to prepare<br>Prolonged separation from family<br>Pet missing or dead<br>Witnessed death/injury to self/ household/ other i.e. neighbor<br>Assisted with rescue and recovery self/ household other i.e. neighbor
Property Hurricane Exposure	Loss of power/electricity<br>Displaced from home<br>Home damaged or destroyed<br>Business damaged or destroyed<br>Vehicle loss<br>Major property loss other than home i.e. furniture<br>Disaster resulted in unemployment (self)<br>Disaster resulted in unemployment (household)<br>Unable to access necessary medical care<br>Unable to access gasoline for vehicle<br>Great difficulty in commuting to work/school due to transportation issues/ gasoline shortage<br>Great difficulty accessing food for self/family<br>Significant financial loss<br>Flooding in home caused by Hurricane Harvey
